# Accelerated Discovery of Cost-Effective Photoabsorber Materials for Near-Infrared (λ = 1600 nm) Photodetector Applications

**DOI:** 10.1021/acsmaterialsau.5c00100

**Published:** 2025-10-20

**Authors:** Wayne Zhao, Ruo Xi Yang, Aaron D. Kaplan, Kristin A. Persson

**Affiliations:** † Department of Materials Science and Engineering, 1438 University of California, Berkeley, California 94720, United States; ‡ Materials Science Division, Lawrence Berkeley National Laboratory, Berkeley, California 94720, United States; ¶ Liquid Sunlight Alliance and Chemical Sciences Division, Lawrence Berkeley National Laboratory, Berkeley, California 94720, United States

**Keywords:** infrared sensing, absorption coefficient, band structure, photodetection, materials discovery, high-throughput screening, computational materials

## Abstract

Current infrared sensing devices are based on costly materials with relatively few viable alternatives known. To identify promising candidate materials for infrared photodetection, we have developed a high-throughput screening methodology based on high-accuracy r^2^SCAN and HSE calculations in density functional theory. Using this method, we identify ten already synthesized materials between the inverse perovskite family, the barium silver pnictide family, the alkaline pnictide family, and ZnSnAs_2_ as top candidates. Among these, ZnSnAs_2_ emerges as the most promising candidate due to its experimentally verified band gap of 0.74 eV at 0 K and its cost-effective synthesis through Bridgman growth. BaAgP also shows potential with an HSE-calculated band gap of 0.64 eV, although further experimental validation is required. Lastly, we discover an additional material, Ca_3_BiP, which has not been previously synthesized, but exhibits a promising optical spectra and a band gap of 0.56 eV. The method applied in this work is sufficiently general to screen wider bandgap materials in high-throughput and now extended to narrow-band gap materials.

## Graphical abstract

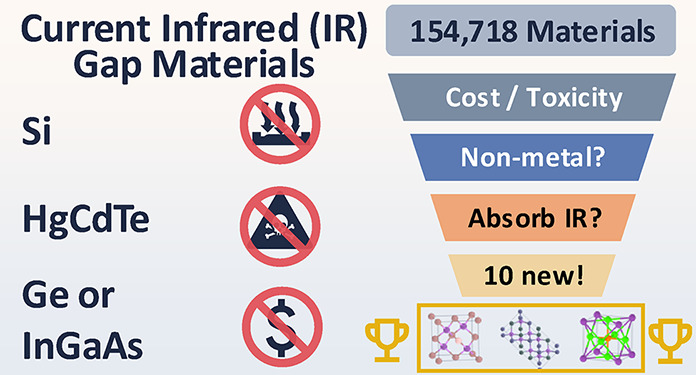

## 1 Introduction

Infrared sensors, such as those in near-infrared (NIR) imaging devices, have diverse applications, such as night vision,^1^ wildfire management,^2^ and spoilage detection in food.^3^ Many cost-effective infrared sensors are silicon-based, which limit detection to wavelengths greater than 1100 nm. However, longer wavelength sensors are essential for detecting a broader range of infrared signals and capturing finer details in imaging. To detect NIR wavelengths up to 1600 nm, a material should exhibit a band gap below 0.77 eV, for example: Ge, which has a band gap of 0.66 eV at 300 K,^4^ and In_0.53_Ga_0.47_As, which has a band gap of 0.75 eV at 300 K.^5^ However, both materials have excessively high production costs that limit widespread use: high purity Ge for NIR sensors has a material feedstock cost 1000 times greater than Si,^6,7^ while In_1–*x*_Ga_*x*_As manufacturing is expensive, partly due to costly growth processes and device packaging.^8^

Other commercially available materials for 1600 nm absorption contain highly toxic or scarce elements such as Hg_1–*x*_Cd_*x*_Te.^9^

There are a few viable alternatives for longer-wavelength IR sensing that have been synthesized and fabricated but not yet commercialized. A relatively nontoxic alternative, GaSb, has a desirable band gap of 0.72 eV^4^ and has been integrated in photodetector devices.^10–12^ Additionally, a previous experimental and theoretical screening of inverse perovskites identified Ca_3_SiO and Ca_3_GeO to be nontoxic and cost-efficient alternatives.^9^ For materials with band gaps outside the NIR energy range, doping to tune band gaps for infrared photodetection is common, such as hyperdoped silicon with gold to create sub-band gap states.^13^ In contrast, it is rarely applied to zero-band gap materials, since there are no sub-band gap states to access. An exception is graphene, but without complex device architectures, the zero band gap of graphene results in high dark current.^14^

To estimate the performance of photodetectors indirectly from materials simulations, Buscema et al. highlighted the importance of using standardized figures-of-merit (FOMs) to compare photodetectors constructed from diverse materials, geometries, and circuit architectures.^15^ A few relevant metrics, such as responsivity, quantum efficiency (QE) and wavelength range, can be indirectly estimated from the electronic structure of the absorbing material. NEP and detectivity most accurately describe photodetector response when accounting for device noise and dark current. Their spectra resemble responsivity and quantum efficiency when signal noise is neglected.^15^ Without reliable signal-to-noise ratio (SNR) data, estimations of NEP and detectivity from materials simulations are beyond the scope of this work. As such, we present responsivity and quantum efficiency as alternative performance metrics that can be computed from materials simulations.

Responsivity and QE are key metrics optimized through device design, reflecting a material’s ability to convert optical power into electrical power. Responsivity and QE for materials like Si, Ge, and In_1–*x*_Ga_*x*_As show a rise in value from short visible wavelengths up to the band gap wavelength-equivalent, beyond which it sharply declines. The absorption edge, defined by the band gap, determines the longest detectable wavelength for photon-based electron–hole pair generation, while the complex dielectric function dictates the absorption coefficient.^16^ For detection beyond silicon’s 1100 nm limit, materials like Ge, In_1–*x*_Ga_*x*_As, or GaSb with suitable band gaps and high absorption coefficients are essential.

These figures of merit demonstrate that the band gap and absorption edge of a material can describe, at a high level, how a material in a simple photodetector would behave before optimizing device architecture. Practical performance metrics, such as high detectivity, high responsivity, and low dark current, may only be realized once implemented in a device. We acknowledge that further optimization—such as targeted doping, alloying, or improved device architecture—may be needed to achieve the highest performance metrics. Thus, this work focuses on computing the band gap and absorption coefficients of materials to estimate the wavelength range and optical behavior of a material in a photodetector. To do so, we combine materials design principles as well as DFT and higher levels of theory to screen materials for applications in NIR sensing. We begin with an overview of the screening parameters used in this work, followed by the computational methods, and last, an in-depth analysis of the most promising candidate materials.

## 2 Methods

### 2.1 Screening

The Materials Project^17,18^ database is surveyed according to a set of physically motivated descriptors for the chosen application. Similar approaches have shown success for solar photocatalysts, photoelectrochemical (PEC) devices, photovoltaic absorbers and transparent conductors for photovoltaic applications.^19–21^ To the best of our knowledge, there have been no systematic high-throughput materials studies targeting longer-wavelength infrared applications.

By design, the initial screening stages are well-established and agreed-upon in the literature, allowing us to build a robust framework for the crucial advances of this work: high-throughput electronic structure determination of solids at the hybrid level of DFT and linear optical response. Note that while there have been a few previous efforts for high-throughput materials exploration with HSE06, e.g., Liu et al.,^22^ these efforts have prioritized thermodynamic properties and band gaps. Our work builds upon this by resolving the full electronic band structure. This approach addresses the challenge of identifying narrow band gap materials and distinguishing semiconducting false metals from true metals, which standard workflows often misclassify.

In the first screening step, shown in Figure 1, materials are selected based on their elemental abundance, toxicity, radioactive stability, PBE band gap, unit cell size, and thermodynamic stability. These materials properties data were taken or derived from the Materials Project^18^ v2023.11.1 data. This screening step drastically reduces the size of the candidate pool from 154,718 to 2951 materials.

**Figure 1:**
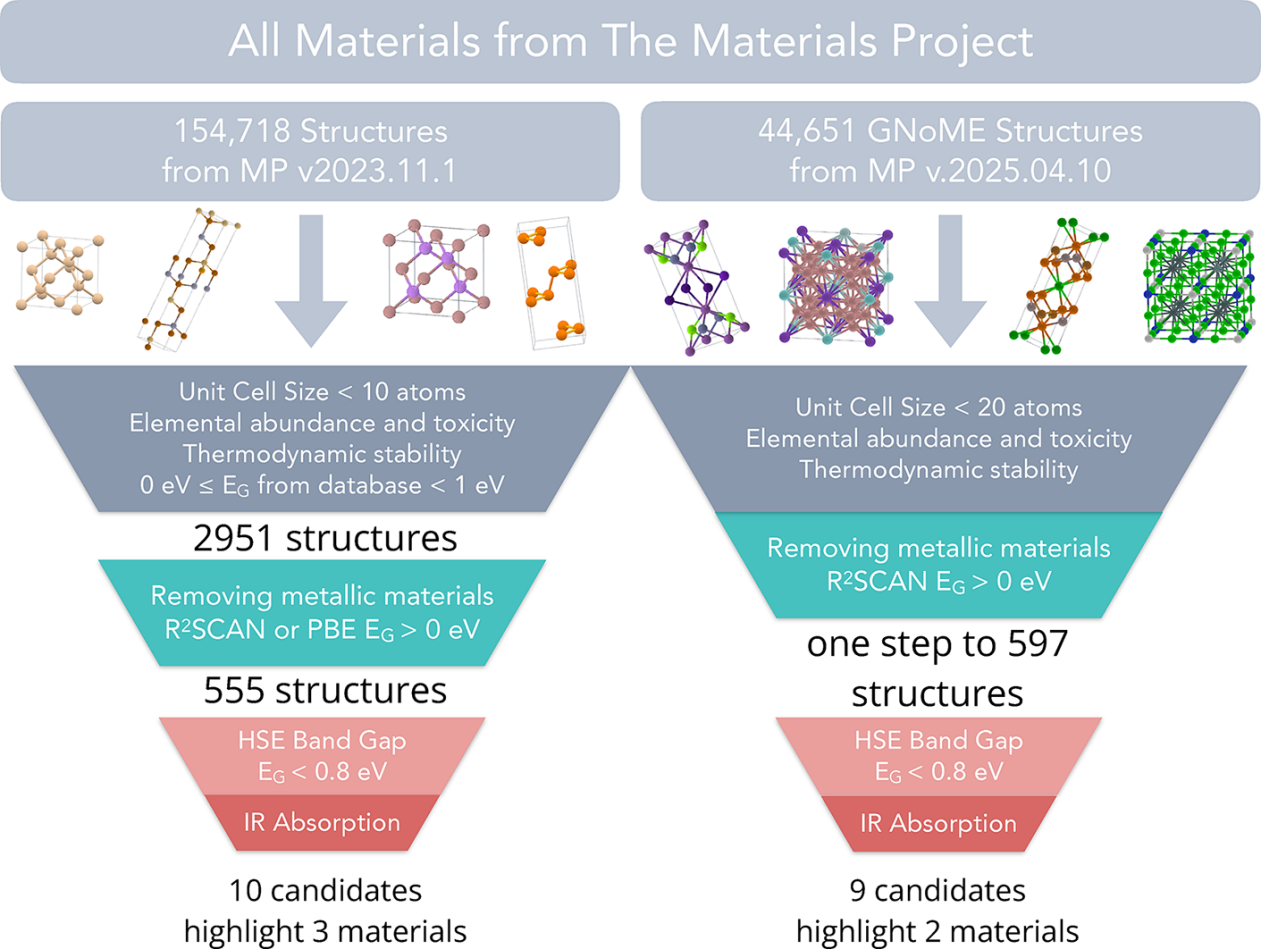
A high-throughput computational screening framework is established, employing criteria ranging from those that are most readily accessible, such as database parsing, to those that are computationally intensive, such as precise and rigorous electronic structure calculations. The left funnel illustrates the screening of materials sourced from version 2023.11.1 of the Materials Project database, while the right funnel corresponds to a subsequent screening of materials derived from the GNoME data set and available through the Materials Project version 2025.04.10. The two data sets are mutually exclusive, sharing no common entries.

When designing low-cost photoabsorbers, ensuring environmental sustainability and human safety during manufacturing and end use is crucial. Drawing from the work of Xiong et al.,^19^ we prioritized elements with a median lethal dose (LD_50_) in excess of 250 mg kg^–1^ and which are labeled as radioactively stable by the International Atomic Energy.^19,23^ It is important to note that elements in unaries can exhibit different toxicity and radioactive stability than in compounds – thus these two screening criteria are intended as a rough approximation of material safety. Thus, arsenic was not excluded by this screening step, as semiconductor end-products such as GaAs or In_1–*x*_Ga_*x*_As are generally considered nonbiologically threatening in passivated wafers,^24^ but exhibit biological risks as pure powder.^24–26^

To estimate material costs, the abundance of elements within the Earth’s crust is used as a heuristic, selecting only elements that have a higher crustal abundance than that of gold at 0.0043 ppm (by mass).^19,27^

Unit cells of candidate materials were strictly limited to ten or fewer atoms to facilitate the calculation of the absorption coefficient.^28^ This size limitation also benefits the computationally expensive band gap calculations used in the latter steps of the screening. For thermodynamic stability, we selected the energy above the thermodynamic hull to be 20 meV or less, chosen as a metric for stability above – 40 °C for typical NIR camera operating temperatures. The cutoff at 20 meV was chosen because thermal energy (*k*_B_ *T*) at this level allows entropy to reduce the free energy.^28^ In oxides or sulfides, entropy can also lead to metastability at energies up to 80 meV.^21^

Most of the materials in the Materials Project include fundamental band gaps calculated using the Perdew–Burke–Ernzerhof (PBE) generalized gradient approximation (GGA).^29^ As it is well-known that PBE tends to underestimate band gaps,^30^ we have considered materials with a PBE bandgap between 0 and 1 eV. This ensures that PBE-predicted false metals, like Ge,^31^ are included. To improve our estimate of narrow-gap semiconductors, we have used bandgaps calculated with the r^2^SCAN meta-GGA^32^ from the Materials Project^33^ in combination with the PBE bandgap: materials with a band gap of 0 eV from both r^2^SCAN and PBE were removed. This choice is justified, e.g., by considering the 2430 insulators in ref 28: of these, 277 were predicted to be metals by PBE but not r^2^SCAN; 29 were predicted metallic by r^2^SCAN but not by PBE; the remaining materials were identified as either metallic, or insulating by both PBE and r^2^SCAN.

In our screening funnel, we rerelaxed the 2951 remaining materials with PBEsol^34^ and then r^2^SCAN. While both PBEsol and r^2^SCAN drastically underestimate bandgaps, they are generally more reliable than hybrid functionals in predicting crystalline geometry.^35^ The remaining 555 materials with a nonzero bandgap were then considered for a more accurate band structure calculation using the Heyd-Scuseria-Ernzerhof 2006 (HSE06) range-separated hybrid-GGA.^36,37^ While higher-level methods, like the random phase approximation (RPA) or Green’s function approaches (GW),^38–40^ could further improve on HSE-level bandgaps,^40^ they are typically too computationally intractable to apply in high throughput. Both approaches require knowledge of unoccupied electronic states, which are typically approximated by ground-state density functionals. Applying either method self-consistently is also extremely challenging, and single-shot calculations may show strong sensitivity to the initial electronic structure. Hybrid density functionals like HSE06 offer comparable accuracy to *GW* theory in predicting electronic band dispersion, and can be more easily applied in high throughput, as they require only the occupied electronic states as input and are relatively easier to apply self-consistently.

All DFT calculations were performed with the Vienna ab initio Simulation Package (VASP),^41–44^ with workflows defined in the atomate2 python package.^45^ To ease self-consistent convergence, the final PBEsol orbitals were used to precondition the r^2^SCAN relaxation. Similarly, the final r^2^SCAN orbitals were used to precondition two HSE06 calculations: a single-point at high *k*-point density for accurate resolution of the electronic density of states, and a line-mode sweep of high-symmetry *k*-points. VASP inputs were based on the MPScanRelaxSet^33^ in pymatgen,^46^ and therefore used a 680 eV plane wave energy cutoff, variable *k*-point density between 0.22 Å^–1^ for materials with an unknown band gap or zero band gap up to 0.44 Å^–1^ for wide-gap insulators,^47^ and energy convergence criterion of 10^–5^ eV. Geometry optimizations were performed until the maximum magnitude of the interatomic forces was below 0.05 eV Å^–1^ for the PBEsol relaxation, and below 0.02 eV Å^–1^ for the r^2^SCAN relaxation. Gaussian Fermi surface broadening was used, with smearing width of 0.05 eV. The “PBE 54” projector augmented wave (PAW) pseudopotentials were used, with specific valence configurations listed in pymatgen. Two HSE06 single-point calculations were then performed: (i) using a high density of *k*-points including two zero-weighted *k*-points at the valence band maximum (VBM) and conduction band minimum (CBM) to improve the bandgap estimate; and (ii) a line-mode sweep of the high-symmetry points in the Brillouin zone following the convention of Setyawan and Curtarolo.^48^ For the high *k*-point density HSE06 static, the tetrahedron integration method with Blöchl corrections was used^49^ to resolve the density of states with high accuracy; for the line-mode sweep, Gaussian smearing of width 0.01 eV was used.

In the development of the workflow for the calculation of the HSE band gaps of materials, a benchmarking study was carried out using the SC40 standard list of semiconductors.^50,51^ This list covers a wide range of zincblende and rocksalt structures, which have been used previously to benchmark HSE band gaps.^50–52^ The workflow in this study demonstrated an accuracy comparable to previous HSE band gap calculations for values exceeding 2 eV, as computed in ref 51. However, for values below 2 eV, the workflow demonstrated improved accuracy (Figure 2), which is especially relevant given this study’s focus on materials with band gaps below 1 eV.

**Figure 2:**
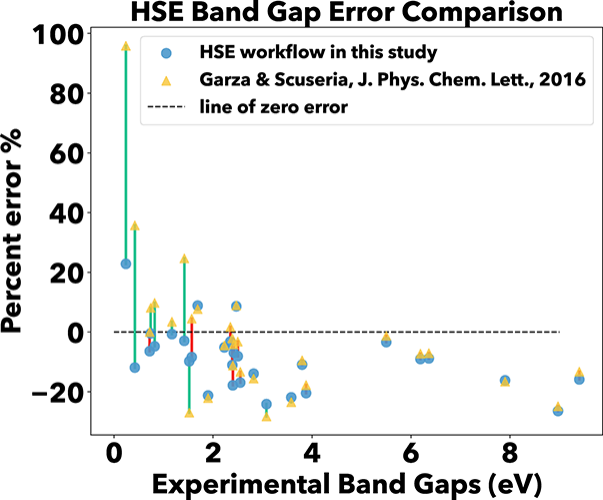
Benchmarking the workflow against previous studies. Yellow triangles represent data from Garza and Scuseria,^51^ while blue circles denote the band gap error obtained in this study. Vertical lines connect band gap errors for the same material; green lines indicate cases where our workflow outperformed previous studies, and red lines indicate the opposite. This benchmarking demonstrates a significant improvement in the accuracy of computing narrow band gap materials below 2 eV.

There are important differences to note between the HSE06 calculations of ref 51 and ours that likely explain this discrepancy at smaller bandgaps. We have used a plane wave basis set code with pseudopotentials, and have chosen to calculate the bandstructure of the structures after optimizing their geometries with r^2^SCAN. Garza and Scuseria^51^ instead used a Gaussian basis set code, with a combination of all-electron calculations for lighter elements, and pseudopotentials (effective core potentials) for heavier elements (as is common practice in Gaussian basis set calculations), and further did not optimize the experimental geometries. While Gaussian orbitals are highly effective for atoms, molecules, and clusters, representing the delocalized electronic densities of periodic solids is more challenging. By contrast, plane-wave bases offer a simple route to systematic convergence via increasing the cutoff energy, whereas commonly used Gaussian sets may exhibit overcompleteness and related linear-dependence or self-consistency convergence issues in solids.^53^ In practice, smaller Gaussian basis sets can introduce modest differences, for example, ref 54 reports band gap deviations on the order of ∼0.05 eV relative to plane-wave calculations, which is consistent with the regions of Figure 2 where the largest discrepancies with ref 51 occur.

We note that HSE06 contains two empirical parameters: the “range-separation cutoff” which controls the interpolation between long-range, semilocal or GGA-like exchange energy, and short-range Hartree–Fock-like exchange, and a linear mixing parameter. Empirically, it has been found^55,56^ that the optimal linear mixing parameter (in our case, that mixing parameter which minimizes errors with respect to experimental bandgaps) corresponds to the inverse of the bulk dielectric constant in solids. Although dielectric-dependent hybrids with self-consistently optimized mixing parameters are well established, e.g., refs 57 and 58, their computational cost makes high-throughput simulations at the scale of this work challenging. Such approaches can improve band gap accuracy, however, we, like many high-throughput studies, trade some rigor for materials coverage and explicitly account for uncertainty when screening candidates.

Following HSE calculations of the band gaps for the 555 materials, any material with a nonzero gap less than 0.8 eV was considered for optical absorption spectrum calculations. The 0.8 eV threshold is derived from the Planck relation, where a photon wavelength of 1600 nm corresponds to a maximum possible band gap of approximately 0.77 eV. This threshold assumes no forbidden transitions at the band gap that would otherwise raise the first allowed transition above the photon energy. GaSb and Ge, with HSE-calculated band gaps of 0.78 and 0.79 eV, respectively, are both established 1600 nm NIR photoabsorbers^5,10^ To account for band gap calculation errors, we apply a buffer to these values, resulting in the 0.8 eV cutoff. This criterion accounts for the fact that semiconducting and insulating materials can exhibit quantum-mechanically forbidden transitions, causing the onset of optical absorption to occur at energies above the band gap energy and increasing the likelihood that a material remains transparent to longer wavelengths of light.^59–61^ The use of a model dielectric function^62^ in predicting absorption near 1600 nm, or a slightly higher bandgap cutoff, may increase the number of NIR-absorbing candidate materials in this tier, and may be explored in future work.

A high-throughput workflow developed by Yang et al.^28^ was used to determine the onset of optical absorption for all candidate materials. This workflow computes the noninteracting or Lindhard dielectric function,^63^ neglecting both random phase approximation (RPA) screening and beyond-RPA local-field effects. To compute the long-wavelength limit of the imaginary part of the frequency-dependent dielectric function,^64^ we used the final orbitals from the r^2^SCAN relaxation to precondition a PBE static calculation. The static calculation used an exact diagonalization of the Hamiltonian to obtain a large number of unoccupied states. The real part was computed via the Kramers–Kronig relation with a 0.1 eV imaginary frequency offset on a regular grid of 2000 real-valued frequency points.

From this screened list of materials, those with infrared absorption characteristics were identified based on a high absorption coefficient of 5 × 10^5^ cm^–1^ and an absorption edge below 0.8 eV, similar to known photoabsorbers such as Ge and GaSb, as illustrated in Figure 3a,b. Materials exhibiting absorption at energies below 0.4 eV were excluded due to increased complexities from thermal noise effects. This leads to increased dark current and increased cost when adding active cooling systems in narrow band gap photodetectors such as the example of HgCdTe.^65^ The materials demonstrating high absorption coefficients at NIR energies are discussed in the following section and listed in Table 1.

**Table 1:** Band Gap Values for Candidate IR Photoabsorbers and Their Respective Material Ids from the Materials Project

formula	known to be NIR absorber	materials project ID	space group	HSE *E*_G_ (eV)
Ge	yes	mp-32	*Fd*3̅*m*1	0.79
GaSb	yes	mp-1156	*F*4̅3*m*	0.78
GaSb	yes	mp-1018059	*P*6_3_ *mc*	0.71
Ca_3_SiO	yes	mp-1205330	*Imma*	0.59
Ca_3_GeO	yes	mp-9721	*Pm*3̅*m*	0.51
Ca_3_GeO	yes	mp-17193	*Imma*	0.68
Ca_3_PbO	no	mp-20273	*Pm*3̅*m*	0.43
Ca_3_BiP	no	mp-1013558	*Pm*3̅*m*	0.56
ZnSnAs_2_	no	mp-5190	*I*4̅2*d*	0.70
BaAgAs	no	mp-7359	*P*6_3_/*mmc*	0.53
BaAgP	no	mp-9899	*P*6_3_/*mmc*	0.64
BaAgSb	no	mp-1205316	*P*6_3_/*mmc*	0.52
KNa_2_Bi	no	mp-863707	*Fm*3̅*m*	0.43
K_3_Bi	no	mp-568516	*Fm*3̅*m*	0.66
K_3_Bi	no	mp-569940	*P*6_3_/*mmc*	0.49
K_2_RbBi	no	mp-1184754	*Fm*3̅*m*	0.69
RbNa_2_Bi	no	mp-1186887	*Fm*3̅*m*	0.42

**Figure 3:**
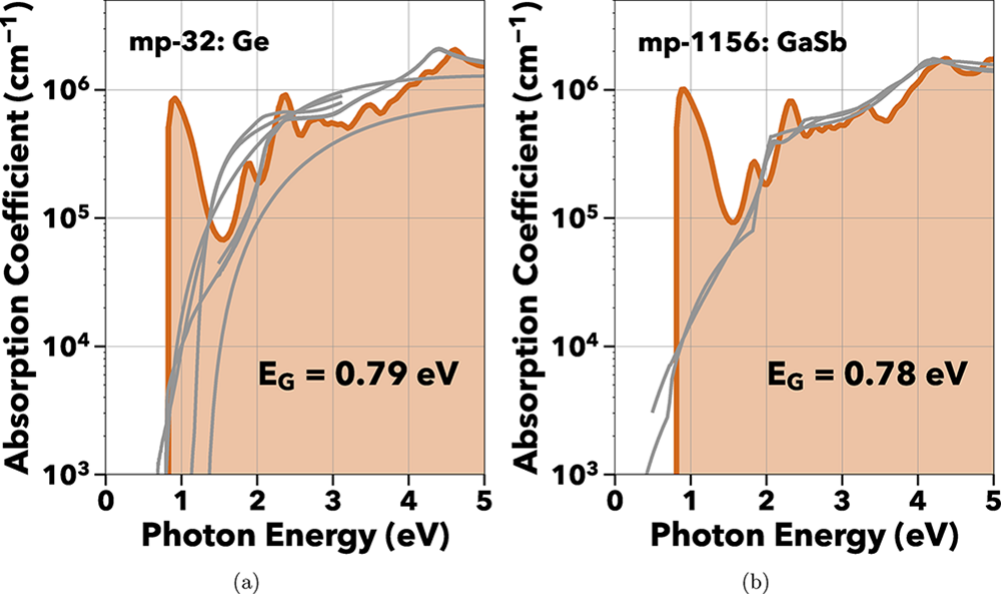
Absorption spectra of experimentally verified photodetector materials for (a) Ge and (b) GaSb. The computed spectra (orange) have high Pearson correlation coefficients of 0.89 for Ge and 0.95 for GaSb, with respect to experimentally determined absorption spectra (gray).^66^

## 3 Results and Discussion

Encouragingly, known IR absorbers such as Ge and GaSb successfully passed the screening, validating the criteria for identifying infrared absorbers. To design novel materials with similar absorption behavior, their spectra should be compared to these known absorbers’ characteristics: a high absorption coefficient value above 1 × 10^5^ cm^–1^ and an absorption edge at the desired photon energy around 0.8 eV.^5^

Figure 3a,b both show steep absorption peaks of ∼1 × 10^6^ cm^–1^ at 0.8 eV. This peak, absent in the experimental data for both Ge and GaSb, arises from the use of PBE orbitals to compute the absorption coefficient in a nonself-consistent manner. PBE predicts both Ge and GaSb to be metallic, leading to high transition rates for electronic excitations near the Fermi level, and thus an incorrectly large absorption coefficient. Despite the presence of this initial peak, the computed spectra of both materials exhibit high Pearson correlation coefficients with respect to experimental spectra.^66^

As a further validation of our methods, two perovskites from ref 9, CaGe_3_O and CaSi_3_O, both emerged from the screening process, although their crystal structures are of the *Imma* symmetry, rather than the slightly distorted *Pnma* symmetry used in ref 9. Consistent with ref 9, Figure 4a,b show strong NIR absorption.

**Figure 4:**
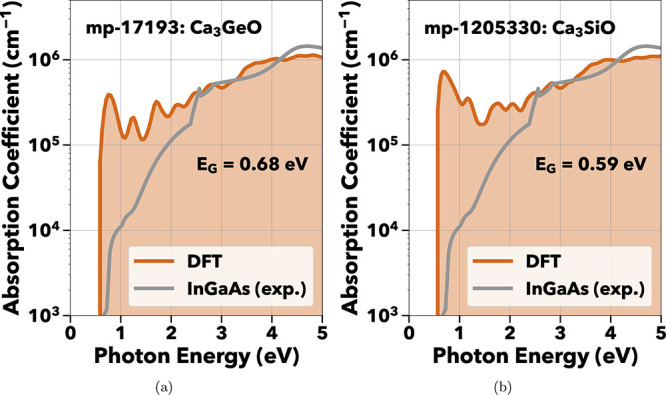
Calculated absorption spectra of experimentally verified inverse-perovskite materials^9^ (a) Ca_3_GeO and (b) Ca_3_SiO. At present, neither have been commercialized nor integrated into photodetectors.

The screening also identified 11 materials which have not been previously considered for NIR applications, but which we found to have prominent NIR absorption: two additional inverse-perovskites, three barium-silver-pnictides, five alkali-bismuthides, and ZnSnAs_2_. We now turn our attention to these 11 candidates, chosen for their high absorption coefficient near 0.8 eV.

An inverse-perovskite, Ca_3_BiP displays strong absorption near 0.6 eV, as shown in Figure 5a. This material has not been previously synthesized, and as such, the toxicity and cost of the precursors are difficult to analyze. Bismuth metal itself costs roughly $12 USD kg^–1^, while phosphate rock costs $0.10 USD kg^–1^ as raw Ca and P feedstock.^7^

**Figure 5:**
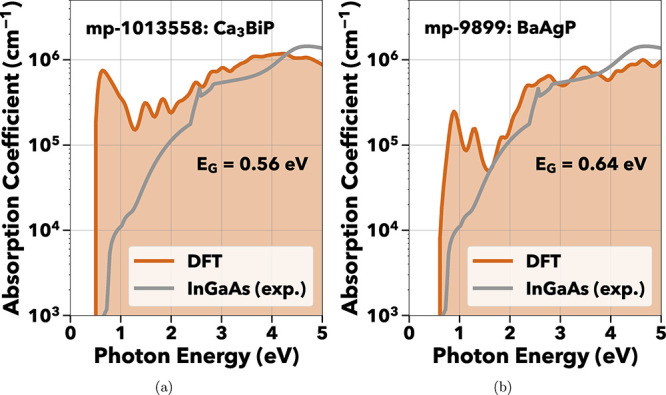
Absorption spectra of Ca_3_BiP and BaAgP both showing predicted absorption in the NIR energies. BaAgP has not been recrystallized while Ca_3_BiP has not been previously synthesized.

Next, we consider BaAgP for its absorption onset at 0.64 eV, as in Figure 5b, and indirect HSE bandgap at the same energy. BaAgP has previously been synthesized in gray metallic powders without recrystallization.^67^ Therefore, BaAgP would need to be recrystallized in a manner similar to BaAgAs,^68^ or grown on a substrate before being implemented in a photodetector.

ZnSnAs_2_, as in Figure 6, crystallizes in both the chalcopyrite and disordered zincblende structures.^69^ First, we discuss the computed optoelectronic properties for the chalcopyrite structure with space group *I*4̅2*d*. The experimentally measured optical energy gap has previously been reported at 0.6–0.66 eV at room temperature and 0.74 eV at 0 K, consistent with the 0.7 eV HSE band gap.^69,70^

**Figure 6:**
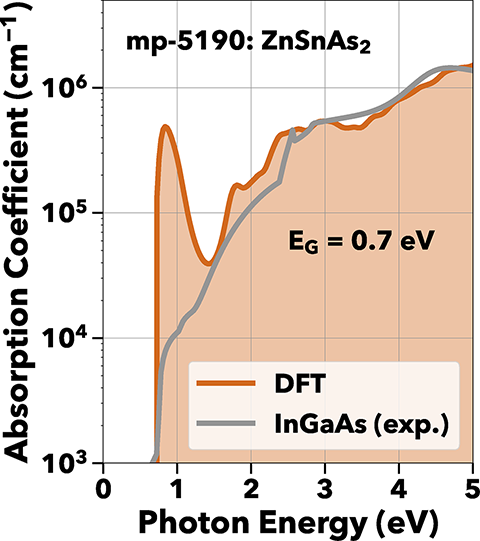
ZnSnAs_2_ shows NIR absorption, albeit with an unphysically large absorption peak similar to Ge and GaSb, due to PBE predicting it to be metallic.

Although this material has not been extensively studied for use as an infrared photodetector, a similar material, ZnSnP_2_, has been shown to absorb up to 850 nm. While the substitution of phosphorus in ZnSnP_2_ for arsenic results in a lower band gap energy, interestingly, ZnSnP_2_ has been shown to exhibit a tunable band gap between 0.75 and 1.75 eV.^71^ The tunability in ZnSnP_2_ is attributed to the degree of cation ordering, whereby - depending on the cooling rates during crystal growth - the ordered chalcopyrite phase transforms into the disordered zincblende phase.^71–74^

We hypothesize that the introduction of (Zn, Sn) cation disorder could lead to a tunable infrared absorbing band gap, similar to that of ZnSnP_2_, and replace toxic but tunable Hg_1–*x*_Cd_*x*_Te currently used photodetector materials. Given that ZnSnP_2_, a member of the same material family, has been successfully used to construct tunable infrared photodetector devices, ZnSnAs_2_ is strongly recommended for further exploration in similar applications.^71^

Crystalline ZnSnAs_2_ can be grown using the relatively cost-effective Bridgman growth technique.^70^ Although arsenic is generally considered toxic, this method combines relatively safe elemental arsenic^75,76^ with zinc and tin pellets, then melts and recrystallizes the mixture to form ZnSnAs_2_ crystals. In contrast, the molecular beam epitaxy (MBE) growth of In_1–*x*_Ga_*x*_As involves high costs and substantial toxicity risks. MBE growth of In_1–*x*_Ga_*x*_As employs the expensive metals indium and gallium, along with the highly toxic arsine gas.^77,78^ Furthermore, ZnSnAs_2_ uses feedstock elements considerably cheaper than those used in current Hg_1–*x*_Cd_*x*_Te alternatives like In_1–*x*_Ga_*x*_As, GaSb, and Ge,^7^ and pose a lower statistical risk to U.S. gross domestic product (GDP) according to the USGS List of Critical Minerals.^79^

With the aim of expanding our search, we computed optical spectra for structures from the GNoME data set^80^ in the Materials Project database. To ensure comprehensive screening, we screened 44,651 GNoME structures with r^2^SCAN data available in Materials Project version 2025.04.10 using the metrics detailed in the Section 2 and illustrated in Figure 1. To demonstrate the robustness of our workflow, we increased the maximum allowed number of atoms per primitive unit cell from ten to 20. Among the screened materials, two candidates, exhibiting near-infrared optical absorption are highlighted: BaSrAg_2_Sb_2_ (Figure 7a) and V_4_GaSe_4_S_4_ (Figure 7b). BaSrAg_2_Sb_2_ has not been synthesized, but is predicted to be an alkaline-silver-pnictide intermetallic. It shares the Zintl-phase ZrBeSi prototype with the previously reported BaAgSb^81^ and SrAgSb.^82^ V_4_GaSe_4_S_4_ has a *F*4̅3*m* crystal structure and the disordered type has been synthesized.^83,84^ Although most GNoME structures lack experimental synthesis data, these two materials possess structural prototypes similar to previously synthesized compounds, which may facilitate their development as near-infrared photoabsorbers.

**Figure 7:**
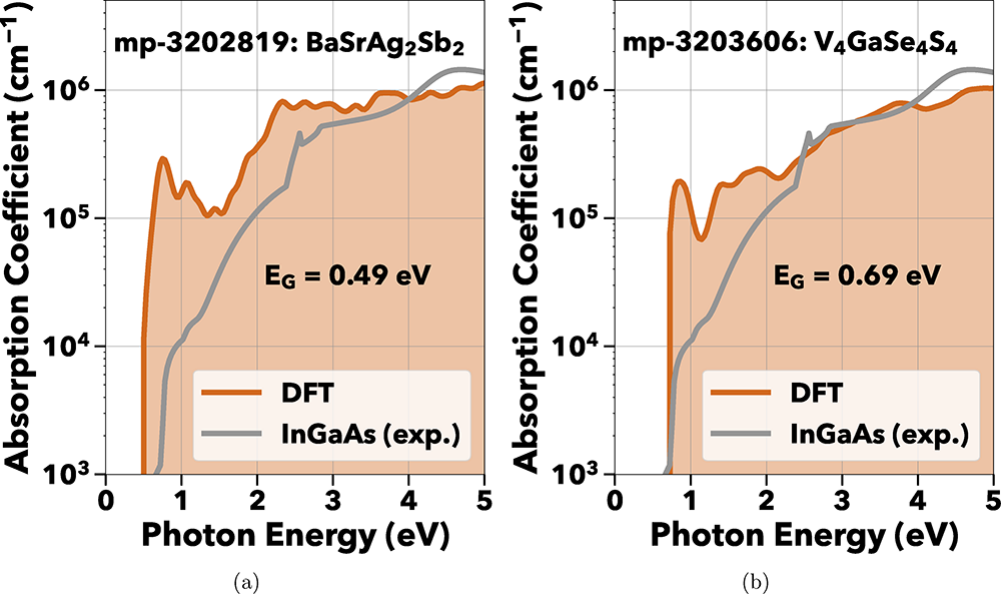
Absorption spectra of BaSrAg_2_Sb_2_ and V_4_GaSe_4_S_4_ both showing predicted absorption in the NIR energies.

## 4 Conclusions

We have developed and tested a high-throughput screening protocol to identify novel near-infrared range (NIR) absorbers. The protocol identified 11 materials, including inverse-perovskites, barium–silver-pnictides, alkaline-pnictides, and ZnSnAs_2_, as NIR absorbers. This adds to previously known NIR absorbers which our protocol also identified: inverse perovskites like Ca_3_SiO, III–V semiconductors like GaSb, and alloys like Hg_1–*x*_Cd_*x*_Te or In_1–*x*_Ga_*x*_As.

ZnSnAs_2_ appears to be the most promising candidate due to its experimentally verified band gap of 0.74 eV at 0 K and its composition of inexpensive elements. ZnSnAs_2_ also shows possible commercial viability as the currently available synthesis method uses relatively safe metal precursors in Bridgman growth, a low-cost and low-toxicity alternative to techniques like molecular beam epitaxy.

We also highlight BaAgP, with an HSE-calculated band gap of 0.64 eV and a high absorption coefficient, though further experimental work is needed to confirm its optical properties and develop a suitable crystal growth technique. Finally, an unsynthesized material, Ca_3_BiP is here predicted to have strong NIR absorbing properties and a desirable band gap.

Additionally, we validate a high-throughput optical absorption workflow^28^ for narrow-gap semiconductors, previously benchmarked against known solar absorbers. We found high correlation with experimental optical spectra, despite occasional false metal classification by PBE. Thus we have demonstrated that PBE orbitals can be used to estimate optical absorption coefficients at the noninteracting electron (Lindhard) level. Our approach represents a computationally tractable, robust, and accurate approach for screening next-generation infrared photoabsorbers.

## Supplementary Material



## Notes

Two promising candidates for middle-wave infrared (MWIR) absorption, inverse-perovskite Ca_3_SnO and alkaline-silver-pnictide family SrAgAs, both of which have band gaps less than 0.4 eV; absorption spectra of candidates not shown in main text; estimation of material costs; and toxicity analysis of arsenic. (PDF)
