# Supplementary material for: Accelerated Discovery of Cost-Effective Photoabsorber Materials for Near-Infrared (λ = 1600 nm) Photodetector Applications
Source: ACS Mater Au. 2025 Oct 20;6(1):119–27. doi: 10.1021/acsmaterialsau.5c00100 (PMC12810037; doi:10.1021/acsmaterialsau.5c00100)
Supplement: Supplementary file 1 [file mg5c00100_si_001.pdf]

Supporting Information: Accelerated discovery of cost-effective photoabsorber materials for near-infrared (λ=1600 nm) photodetector applications

Wayne Zhao,[†,‡,¶] Ruo Xi Yang,[‡,§] Aaron D. Kaplan,[‡,§] and Kristin A. Persson[*,†,‡,¶]

†*Department of Materials Science and Engineering, University of California, Berkeley, Berkeley, CA, USA 94720*

‡*Materials Science Division, Lawrence Berkeley National Laboratory, Berkeley, CA, USA 94720*

¶*Liquid Sunlight Alliance and Chemical Sciences Division, Lawrence Berkeley National Laboratory, Berkeley, 94720, CA, USA*

§*These authors contributed equally to this work.*

E-mail: kristinpersson@berkeley.edu

Additional inverse-perovskites

Ca_3SnO and Ca_3PbO have small band gaps of 0.39 eV and 0.43 eV, respectively, and are promising for middle wavelength infrared (MWIR) detection. Their absorption coefficients are plotted in Fig. S1a and Fig. S1b. We show that the substitution of the p-block element M in the R_3MO composition results in relatively minimal variation in the computed band

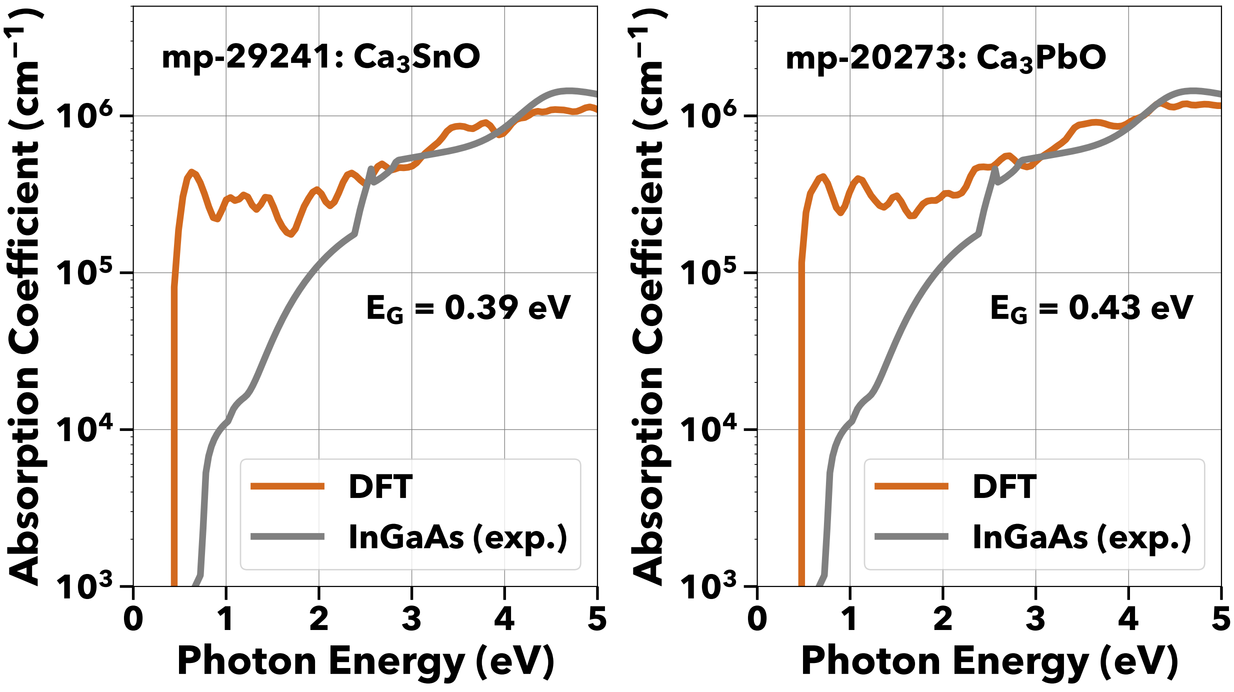

(a) (b)

Figure S1: Absorption spectra of additional inverse perovskite materials (a) Ca_3PbO and (b) Ca_3SnO.

gap. Among the considered compositions, Ca_3SiO has the largest band gap at 0.59 eV, while Ca_3SnO has the smallest band gap at 0.39 eV.

Additional barium silver pnictides

Materials BaAgAs and BaAgSb also emerged as candidates of the ZrBeSi-type Zintl-phase[S1–S3] . Both of these materials have been synthesized and/or studied as materials for thermoelectric applications, but their optoelectronic properties for infrared absorption have not been experimentally tested[S4,S5].

BaAgAs shows high absorption coefficients and a HSE computed band gap of 0.53 eV. BaAgAs has been experimentally synthesized and grown as a crystal, although XRD indicated that their sample may have been partially non-crystalline[S4]. Xu *et al.*[S4] showed that BaAgAs is metallic, albeit with resistivity increasing with temperature from 0 K to 300 K.

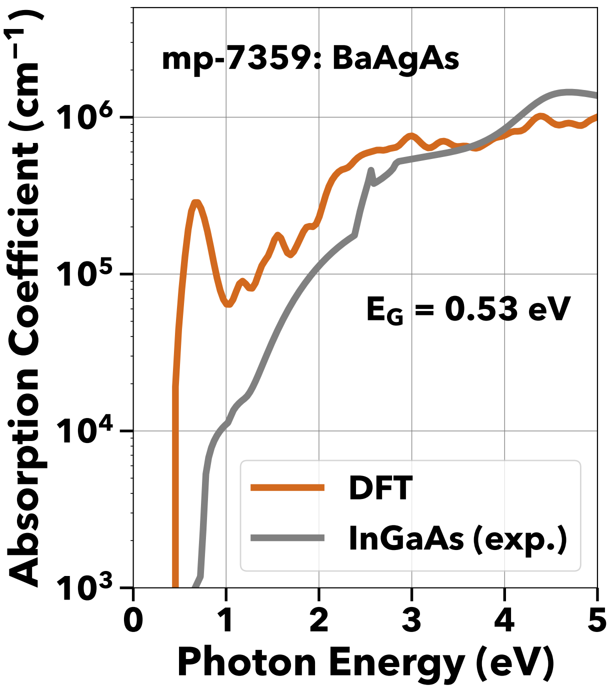

(a)

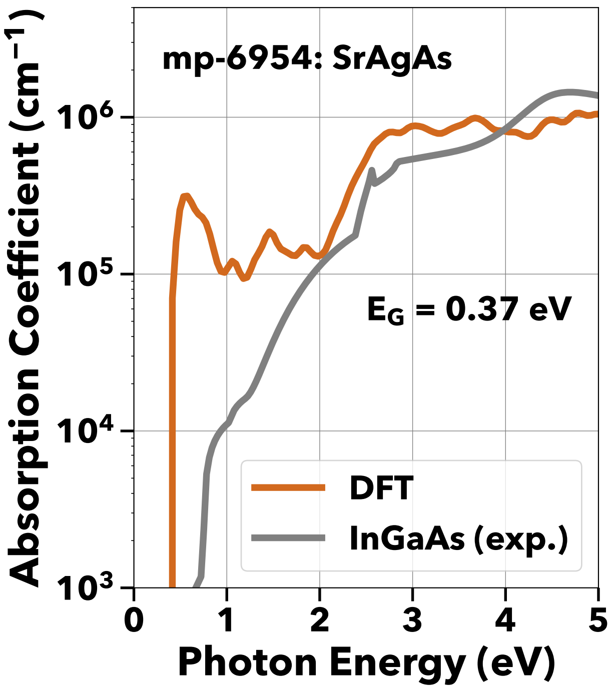

(b)

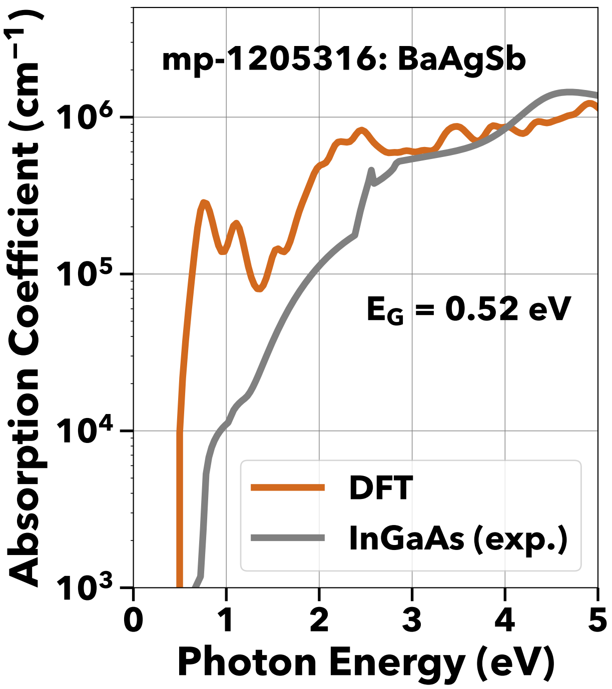

(c)

Figure S2: Absorption spectra of BaAgAs and BaAgSb with the predicted optoelectronic properties of infrared light absorption. Additionally, we predict SrAgAs as a narrow band gap photoabsorber for MWIR absorption.

In the same crystal structure, SrAgAs emerged as a potential MWIR photoabsorber with a low direct band gap of 0.37 eV.

BaAgSb was predicted to have an HSE band gap of 0.52 eV and shows a high absorption coefficient. Few studies have been published on the synthesis and characterization of BaAgSb. Huang *et al.* measured the optical band gap of BaAgSb to be 0.13 eV at the specific chemical composition of $Ba_{0.98}AgSb$[S5]. Additional experimental studies analyzing the optoelectronic properties of this material at stoichiometric ratios are necessary to verify the band gap as the small 0.13 eV band gap may be due to off-stoichiometry defect states.

Alkali bismuthides

Alkali pnictides, including alkali antimonides, alkali bismuthides, and bialkali antimonides, have attracted significant interest for their potential application in optoelectronic devices such as photodetectors[S6,S7]. The present study has identified four alkali bismuthides that have been deemed novel, mainly because of a dearth of experimental data. However, computational results indicate that these materials exhibit robust absorption in the near-infrared region.

K_3Bi shows a steep absorption edge at 0.49 eV and shows absorption at the desired 0.8 eV. K_3Bi has been synthesized using potassium and bismuth through molecular beam epitaxy (MBE) in ultra-high vacuum conditions to avoid the handling difficulties associated with its high chemical reactivity in air[S8]. Wen *et al.*[S8] reported that K_3Bi is highly air sensitive, which requires an inert atmosphere during production and additional packaging to prevent degradation, thus significantly increasing manufacturing costs. Angle-resolved photoemission spectroscopy (ARPES) measurements have revealed that K_3Bi is a Dirac semi-metal, presenting additional challenges for its practical use[S8]. Although K_3Bi can be grown on Na_3Bi-Si substrates, which could facilitate integration into silicon-based sensors, it is recommended for further study for its lower air stability and experimental measurements as a semi-metal.

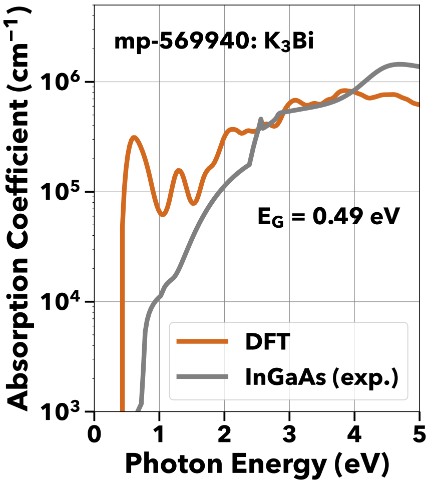
mp-569940: K3Bi
EG = 0.49 eV
DFT
InGaAs (exp.)
Absorption Coefficient (cm−1)
Photon Energy (eV)

(a)

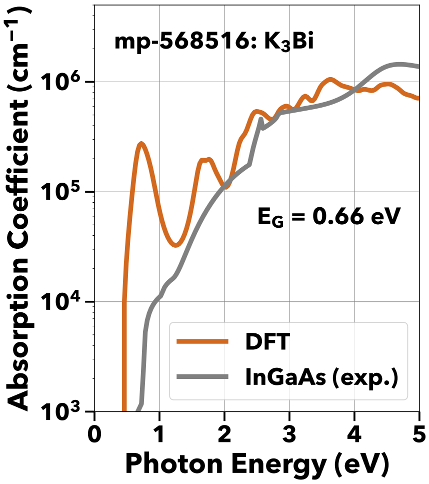
mp-568516: K3Bi
EG = 0.66 eV
DFT
InGaAs (exp.)
Absorption Coefficient (cm−1)
Photon Energy (eV)

(b)

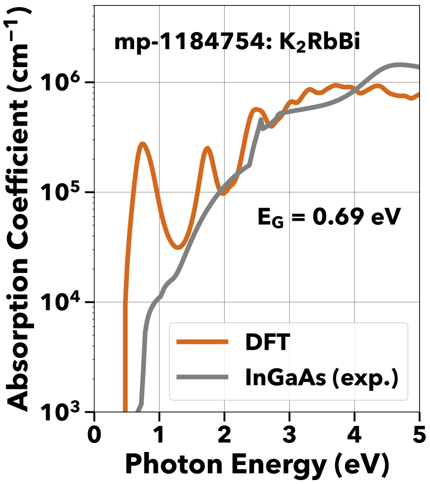
mp-1184754: K2RbBi
EG = 0.69 eV
DFT
InGaAs (exp.)
Absorption Coefficient (cm−1)
Photon Energy (eV)

(c)

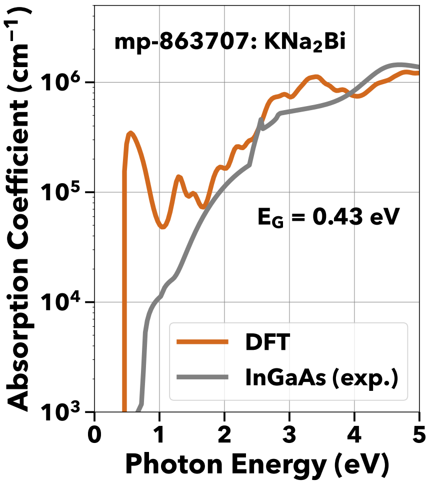
mp-863707: KNa2Bi
EG = 0.43 eV
DFT
InGaAs (exp.)
Absorption Coefficient (cm−1)
Photon Energy (eV)

(d)

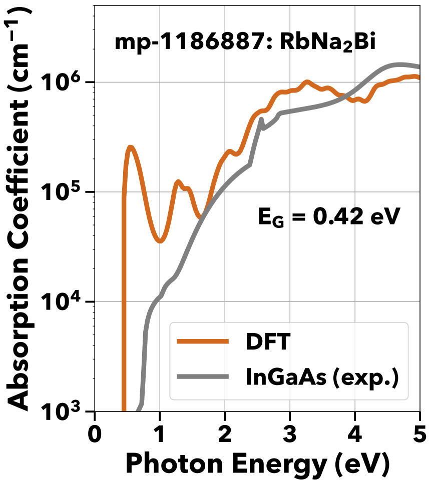
mp-1186887: RbNa2Bi
EG = 0.42 eV
DFT
InGaAs (exp.)
Absorption Coefficient (cm−1)
Photon Energy (eV)

(e)

K_2RbBi, $RbNa_2Bi$, KNa_2Bi all show absorption at the desired 0.8 eV energy. However, there is limited literature, particularly experimental data, on the optical behavior of these materials. KNa_2Bi has been computationally predicted to be a topological insulator under pressure[S9]. These materials may likely be semimetals as alkali-pnictides in the chemical composition Ak_3Pn have been predicted to show band inversion as Dirac semimetals[S10].

Estimates of Material Costs

Using data from the United States Geological Survey,[S11] we have estimated the rough cost of a compound from elemental feedstock costs. These costs are shown in Fig. S3. It should be noted that compounds are not always synthesized from unary reactants, and are often synthesized from compound reactants. Thus, the extraction of a pure unary from a compound form may add artificial cost to our estimate. However, in the absence of a library of synthesis recipes and precise, stable cost estimates for compound reactants, we find the estimates of Fig. S3 to be reasonable.

Comparison to $In_{1-x}Ga_xAs$

We evaluated the candidate materials by comparing their predicted optical absorption spectra with the experimental spectrum of the benchmark material, $In_{1-x}Ga_xAs$.[S12] In Fig. S4, we present comparisons for the three materials featured in the main article, as well as an additional material that, although it does not exhibit a high predicted onset absorption, closely resembles the absorption curve of $In_{1-x}Ga_xAs$ with a computed HSE electronic band gap of 0.52 eV.

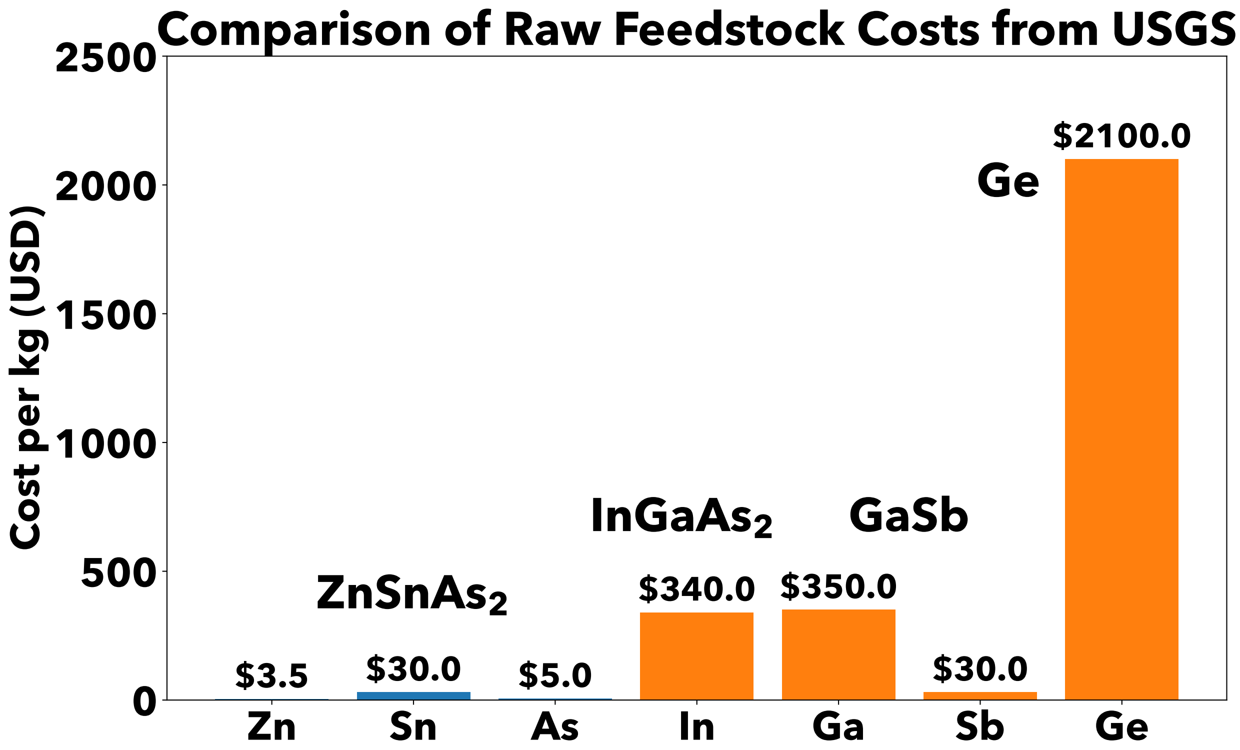

Figure S3: Cost of raw elements used in infrared light detection according to the United States Geological Survey 2025 Mineral Commodity Summary[S11].

Toxicity of Arsenic

In the context of semiconductor growth, elemental arsenic—used in the Bridgman growth process of $ZnSnAs_2$—is reported to have relatively low toxicity, with few established quantitative limits[S13,S14]. In contrast, arsine gas is highly toxic, with a reported lethal concentration of 250 ppm[S15]. While toxicity is a multifaceted issue warranting further toxicological research on the candidate materials themselves, we use the toxicity of precursor substances as a heuristic to estimate potential human health risks, as well as associated manufacturing costs related to safety measures and regulatory compliance. A detailed toxicological assessment is beyond the scope of this study; however, prior work has highlighted the complexities of human arsenic metabolism[S16] and has consistently shown arsine to be far more toxic than elemental arsenic[S14,S15]

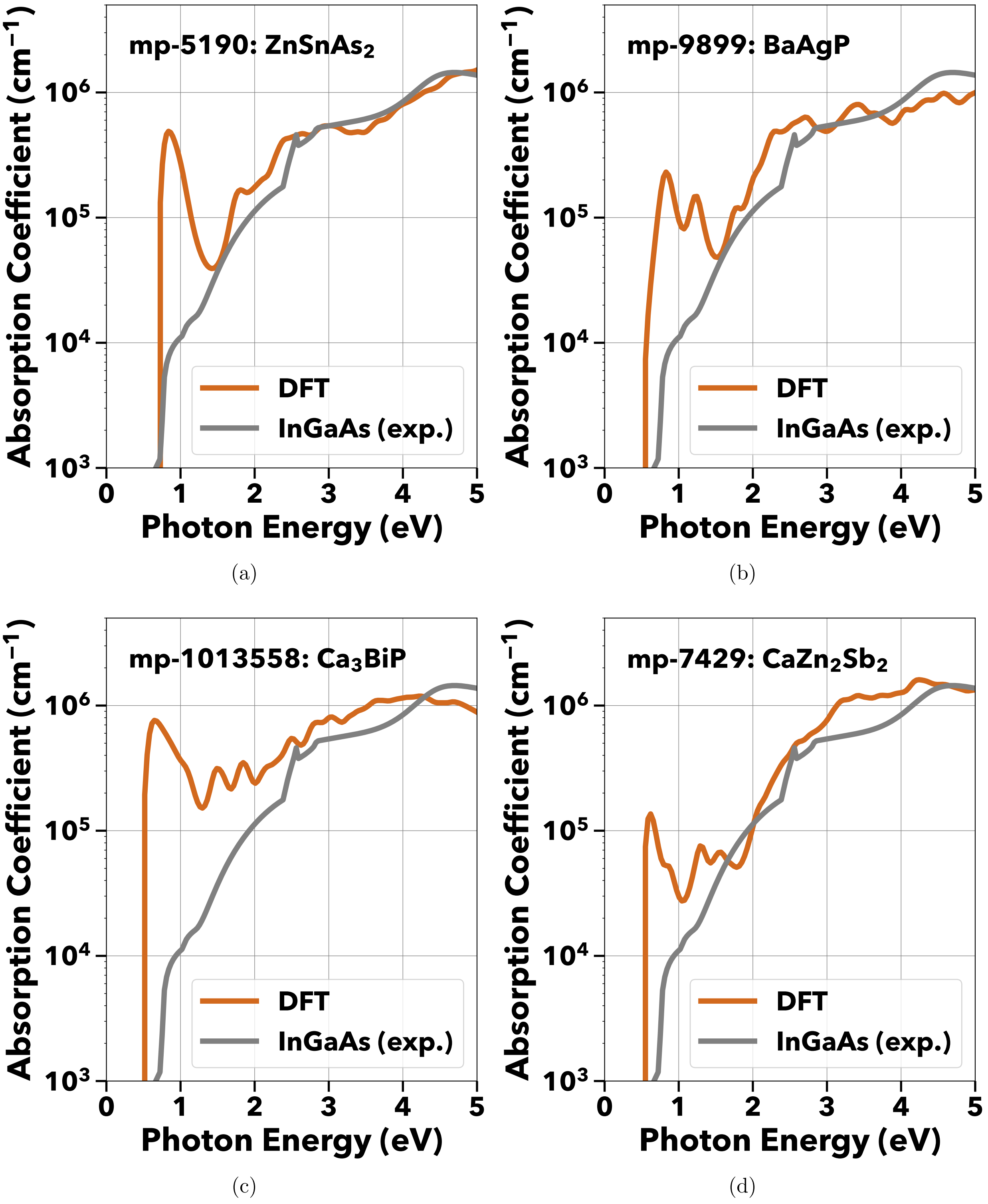

Figure S4: Absorption spectra of $ZnSnAs_2$, BaAgP, Ca_3BiP and $ZaZn_2Sb_2$ plotted against experimental $In_{1-x}Ga_xAs$ experimental data.

References

(S1) Parvin, F.; Hossain, M.; Ahmed, I.; Akter, K.; Islam, A. First-principles calculations to investigate mechanical, optoelectronic and thermoelectric properties of half-Heusler p-type semiconductor BaAgP. *Results in Physics* **2021**, *23*, 104068.

(S2) Wang, S.-F.; Zhang, Z.-G.; Wang, B.-T.; Zhang, J.-R.; Wang, F.-W. Zintl phase BaAgSb: Low thermal conductivity and high performance thermoelectric material in ab initio calculation. *Chinese Physics Letters* **2021**, *38*, 046301.

(S3) Zhang, W.; Chen, C.; Yao, H.; Xue, W.; Li, S.; Bai, F.; Huang, Y.; Li, X.; Lin, X.; Cao, F.; others Promising Zintl-phase thermoelectric compound SrAgSb. *Chemistry of Materials* **2020**, *32*, 6983–6989.

(S4) Xu, S.; Wang, H.; Wang, Y.-Y.; Su, Y.; Wang, X.-Y.; Xia, T.-L. Crystal growth of BaAgAs family topological materials via flux method. *Journal of Crystal Growth* **2020**, *531*, 125304.

(S5) Huang, Y.; Chen, C.; Zhang, W.; Li, X.; Xue, W.; Wang, X.; Liu, Y.; Yao, H.; Zhang, Z.; Chen, Y.; others Point defect approach to enhance the thermoelectric performance of Zintl-phase BaAgSb. *Science China Materials* **2021**, *64*, 2541–2550.

(S6) Spicer, W. E. Photoemissive, Photoconductive, and Optical Absorption Studies of Alkali-Antimony Compounds. *Phys. Rev.* **1958**, *112*, 114–122.

(S7) Yalameha, S.; Nourbakhsh, Z.; Ramazani, A.; Vashaee, D. Promising Bialkali Bismuthides Cs(Na, K)2Bi for High-Performance Nanoscale Electromechanical Devices: Prediction of Mechanical and Anisotropic Elastic Properties under Hydrostatic Tension and Compression and Tunable Auxetic Properties. *Nanomaterials* **2021**, *11*.

(S8) Wen, J.; Guo, H.; Yan, C.-H.; Wang, Z.-Y.; Chang, K.; Deng, P.; Zhang, T.; Zhang, Z.-

D.; Ji, S.-H.; Wang, L.-L.; others Synthesis of semimetal A3Bi (A= Na, K) thin films by molecular beam epitaxy. *Applied Surface Science* **2015**, *327*, 213–217.

(S9) Sklyadneva, I. Y.; Rusinov, I. P.; Heid, R.; Bohnen, K.-P.; Echenique, P. M.; Chulkov, E. V. Pressure-induced topological phases of KNa2Bi. *Scientific Reports* **2016**, *6*, 24137.

(S10) Armitage, N. P.; Mele, E. J.; Vishwanath, A. Weyl and Dirac semimetals in three-dimensional solids. *Rev. Mod. Phys.* **2018**, *90*, 015001.

(S11) U.S. Geological Survey *Mineral Commodity Summaries 2025*; Report 2025, 2025; p 212.

(S12) Polyanskiy, M. N. Refractiveindex.info database of optical constants. *Scientific Data* **2024**, *11*, 94.

(S13) Peters, G. R.; McCurdy, R. F.; Hindmarsh, J. T. Environmental aspects of arsenic toxicity. *Critical Reviews in Clinical Laboratory Sciences* **1996**, *33*, 457–493.

(S14) Danielson, C.; Houseworth, J.; Skipworth, E.; Smith, D.; McCarthy, L.; Nanagas, K. Arsine toxicity treated with red blood cell and plasma exchanges. *Transfusion* **2006**, *46*, 1576–1579.

(S15) Pakulska, D.; Czerczak, S. Hazardous effects of arsine: a short review. *International Journal of Occupational Medicine and Environmental Health* **2006**, *19*, 36–44.

(S16) Carter, D. E.; Aposhian, H. V.; Gandolfi, A. J. The metabolism of inorganic arsenic oxides, gallium arsenide, and arsine: a toxicochemical review. *Toxicology and Applied pharmacology* **2003**, *193*, 309–334.
